# Response of Barley Plants to Drought Might Be Associated with the Recruiting of Soil-Borne Endophytes

**DOI:** 10.3390/microorganisms8091414

**Published:** 2020-09-14

**Authors:** Luhua Yang, Peter Schröder, Gisle Vestergaard, Michael Schloter, Viviane Radl

**Affiliations:** 1 Helmholtz Zentrum München, Research Unit Comparative Microbiome Analysis, Ingolstädterlandstr. 1, 85764 Oberschleißheim, Germany; luhua.yang1987@gmail.com (L.Y.); peter.schroeder@helmholtz-muenchen.de (P.S.); schloter@helmholtz-muenchen.de (M.S.); 2 Section for Bioinformatics, Department of Health Technology, Technical University of Denmark, DK-2800 Lyngby, Denmark; gisves@dtu.dk

**Keywords:** abiotic stress, drought, microbiome, diversity, root endophytes, environmental factors

## Abstract

Mechanisms used by plants to respond to water limitation have been extensively studied. However, even though the inoculation of beneficial microbes has been shown to improve plant performance under drought stress, the inherent role of soil microbes on plant response has been less considered. In the present work, we assessed the importance of the soil microbiome for the growth of barley plants under drought stress. Plant growth was not significantly affected by the disturbance of the soil microbiome under regular watering. However, after drought stress, we observed a significant reduction in plant biomass, particularly of the root system. Plants grown in the soil with disturbed microbiome were significantly more affected by drought and did not recover two weeks after re-watering. These effects were accompanied by changes in the composition of endophytic fungal and bacterial communities. Under natural conditions, soil-derived plant endophytes were major colonizers of plant roots, such as Glycomyces and Fusarium, whereas, for plants grown in the soil with disturbed microbiome seed-born bacterial endophytes, e.g., Pantoea, Erwinia, and unclassified Pseudomonaceae and fungal genera normally associated with pathogenesis, such as Gibberella and Gaeumannomyces were observed. Therefore, the role of the composition of the indigenous soil microbiota should be considered in future approaches to develop management strategies to make plants more resistant towards abiotic stress, such as drought.

## 1. Introduction

Due to their sessile lifestyle, plants are directly exposed to changing environmental conditions, which could have a strong impact on their survival. In agricultural systems, this could lead to losses in productivity, representing a major constraint for food security. In fact, the increase of extreme weather events, including drought, heat, and flooding, is already affecting food production on a global scale. For cereal crops, yield reductions of 9 to 10% have been documented in the last decades [1]. 

Water limitation affects seed germination, establishment of seedlings and subsequent vegetative growth. The influence on later plant performance and yield depends both on the exact time point of the drought during plant development and the duration. Drought stress can lead to poor germination rates due to reduced water uptake during the imbibition phase, reduced energy supply, and impaired enzyme activity [2]. Furthermore, water stress might interfere with the vegetative growth, nutrient acquisition, photosynthesis rates and assimilate partitioning of growing plants [3]. Plants developed different strategies to handle drought stress during evolution [2]. To avoid dehydration, plants can reduce water losses via transpiration and increase water uptake by the roots. Stomatal closure is one of the first responses of plants to drought [4], which leads to a decline of leaf internal CO_2_ and, hence, photosynthesis [5]. On the other hand, plants with better ability to extract water from the soil are more resistant to drought. This is achieved via changes in root morphology [6]. In addition, tolerance to drought stress can be also accomplished by physiological adaptations. For example, plants can regulate their turgor pressure under drought stress by increasing the concentration of compatible solutes, such as sugars, sugar alcohols, proline, and glycinebetain [7]. Besides, the enhanced production of antioxidative enzymes is also a crucial response to drought stress, as under limited water availability reactive oxygen species are produced, which can affect cell metabolism [8]. 

Even though microbes have been shown to improve plant performance under drought stress [9], bacteria, fungi, and archaea have been scarcely considered in theories about mechanisms by which plants respond or even adapt to water limitation. Inoculation of plant seeds with probiotic microbiota has increased germination rates, changed plant root architecture, improved reactive oxygen species (ROS)response, and raised the concentration of proteins and sugars as well as enhanced proline contents in leaves [10,11,12]. Moreover, as shown by Castillo et al. [13] the inoculation of seedlings with selected bacterial strains modifies plant hormone balance, for example, by increasing the salicylic acid concentrations in shoots. In addition, the improved performance of pea plants under drought stress due to the inoculation with a *Pseudomonas* sp. strain producing 1-aminocyclopropane-1-carboxylic acid (ACC)-deaminase was explained by a reduction of ethylene levels in the plant tissue [14]. The inoculation of plants with microbes may induce several changes in the plant phenotype. For example, Pandey et al. [15] demonstrated the role of *Trichoderma harzianum* on the mitigation of drought stress in rice plants due to the upregulation of aquaporin, dehydrin and malonialdehyde. Yet most studies, showing the importance of microorganisms for improved stress responses of plants, are based on inoculation experiments using single bacterial or fungal strains and do not take into account the role of the soil microbiome in the mitigation of drought stress in plants. 

A study of Lau and Lennon [16] gave first evidence for the important role of the soil microbiome for the fast adaptation of plants to drought. The authors demonstrated that plants grown in soils, which harbor microbial communities pre-conditioned to drought, performed better when facing drought than those grown in soils with a non-adapted microbiome. Likewise, it was shown recently that plant–soil feedbacks can be affected by legacy effects of drought on soil microbes [17]. 

However, the inherent role of the soil microbiome on the response of plants to drought stress still needs to be addressed. Therefore, in the present study, we carried out a pot experiment where the fitness of barley plants under optimal growth conditions and drought stress was compared when plants were grown in an arable soil in its natural status or with a disturbed microbiome as a result of repeated autoclaving. We investigated plant responses directly after a drought setting and after a regeneration period under normal water conditions. We linked plant responses to the diversity of root endophytes, which are considered as an important group of microbes driving plant stress response [18]. In the respective treatments, we assessed how the composition of root endophytes was affected by drought in both soils using a molecular barcoding approach for bacteria and fungi. We postulated that plants have a comparable performance in both soils under optimal growth conditions. However, once subjected to drought stress, plants grown in soil with natural microbial communities will perform better compared to plants grown in soil with a disturbed microbiome, which indicates the importance of soil born microbes as part of the endophytic microbiome.

## 2. Material and Methods

### 2.1. Experimental Design

In the present study we investigated how soil microbes influence the response of barley plants to drought stress. Therefore, plants grown in an agricultural soil holding its natural or a disturbed microbial community were submitted to drought. Changes in soil properties after sterilization is a well-known phenomenon [19]. Additionally, nutrient release due to cell lyses could influence plant growth [20]. Therefore, we have performed pre-experiments to adjust ideal watering conditions and the time and the extent of water stress. Moreover, we did fertilize the soil (below) to minimize effects on plant growth, which were not directly caused by the disturbance of microbial communities.

We used soil from a Research Farm located in Scheyern, Germany (48°30′05″ N, 11°27′10″ E). Top soil (0–20 cm) was taken after the harvest of rapeseed. The soil was classified as a Luvisol (World Reference Base for Soil Resources), with sandy loamy texture (43% sand, 33% silt and 24% clay). The soil was sieved through a 2 mm mesh and divided into two portions. One portion of the soil was stored at room temperature for approximately 10 days, labeled throughout the text as soil with natural microbiome (NSM). The other portion was autoclaved at 134 °C for 2 h twice with an interval of 7 days and was labeled as autoclaved soil with disturbed microbiome (DSM). This procedure was done to reduce soil microbial biomass. Serial dilutions of soil extracts plated in R2A media detected 10^7^ cells. g soil^−1^ in NSM soil, but no growth in DSM. Moreover, to confirm the reduction of microbial biomass, cells from both NSM and DSM were extracted according to Eichorst et al. [21]. The cells were fixed with 4% Paraformaldehyde, stained with 4′,6-diamidino-2-phenylindole (DAPI) and checked using a fluorescence microscope (Zeiss, Oberkochen, Germany). As expected, we observed a clear reduction in cell numbers in the autoclaved soil.

The *Hordeum vulgare* L. cultivar Barke used in this experiment was kindly provided by Saatzucht Breun GmbH and Co. KG, Herzogenaurach, Germany. To avoid growth of contaminating microbes from the seed surface in the experiment, seeds were surface sterilized. Therefore, seeds were immersed in 1% Tween 20 for 2 min and in 70% ethanol for 5 min and washed five times with sterile water prior to germination. Seeds were germinated under sterile conditions in Petri dishes containing humidified filter papers at room temperature in the dark for 72 h. Germinated seeds were transferred to pots (13 × 13 × 13 cm) filled with 1.5 kg NSM and DSM soil, respectively. Each pot contained two germinated seeds. The plants were grown in greenhouse under controlled conditions with 12 h light at 22 °C and 12 h dark at 18 °C. The water content of the soil was adjusted to approximately 60% of the maximal water holding capacity, which corresponded to 27% ± 0.01 and 25% ± 0.01 for NSM and DSM, respectively, measured using a gravimetric method. In the first two months, all barley plants were equally treated. Plants were irrigated with 200 mL tap water twice per week to adjust the water content. Plants were fertilized once a week by adding 200 mL one-tenth Hoagland solution (Sigma-Aldrich, St. Louis, MO, USA).

Two months after sowing (Zadok growth stage scale 34), we imposed drought to plants grown in the NSM (labeled as NSM-D1 and NSM-D2) and DSM (labelled as DSM-D), by completely ceasing irrigation for 11 days. Corresponding control samples (labelled as NSM-C, DSM-C) were watered regularly thrice a week without additional fertilization. 

**Figure 1:**
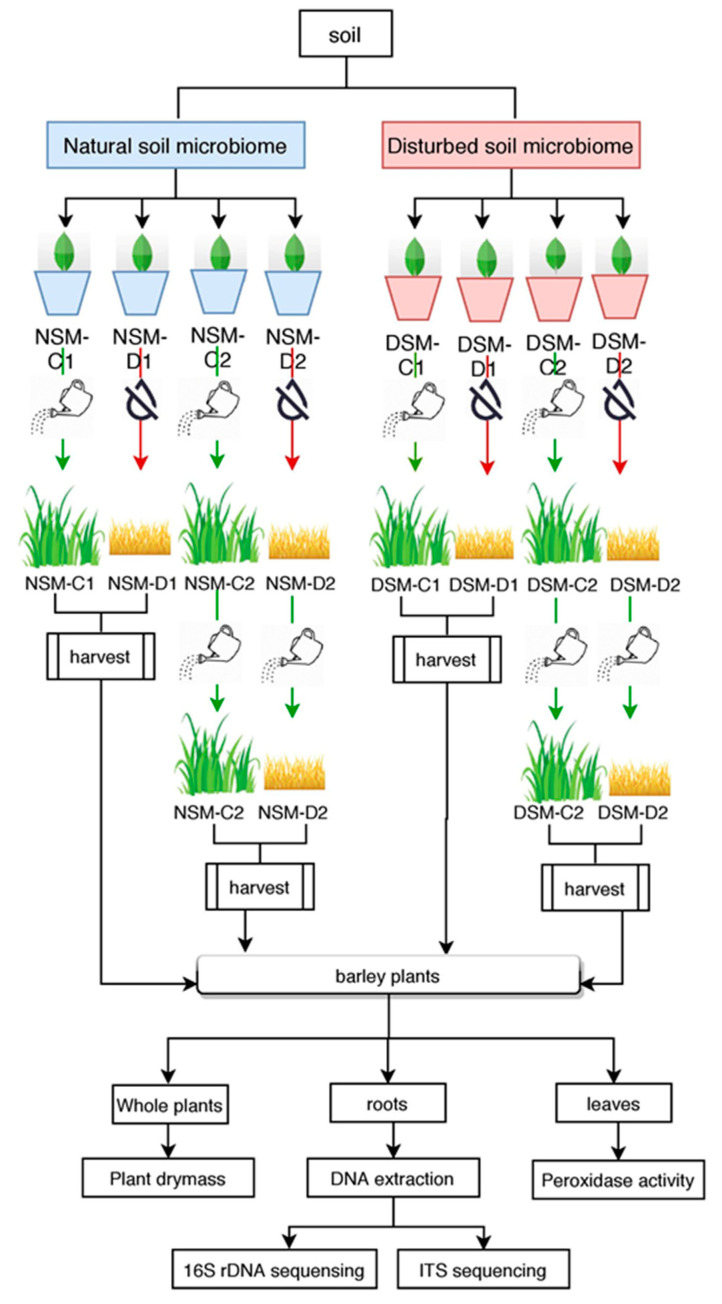
Detailed outline of the experiment design.

We performed destructive sampling at two time points: (i) T1, 11 days after last watering (NSM-D1, NSM-D1, NSM-C1 and DSM-C1) and (ii) T2, 14 days after plants were re-watered (NSM-D2, NSM-D2, NSM-C2 and DSM-C2). For each treatment, 13 replicates were prepared. All pots were randomly placed in the green house. Figure 1 illustrates a detailed outline of the experiment design.

### 2.2. Plants Harvesting

Plants were carefully removed from the pots to avoid the rupture of the roots. The attached soil was briefly shaken off. Five randomly selected plants from each treatment were kept intact. They were washed with water to remove attached soil. The intact clean plants were then cut into roots and leaves. The samples were oven-dried at 105 °C for 72 h. The dry mass of barley leaves and roots were measured gravimetrically. The total plant dry mass was calculated as the sum of barley leaves and roots.

Leaves and roots of the remaining plants (8 replicates) were separated using sterile scalpels. The leaves were immediately frozen in liquid nitrogen and stored at −80 °C for further biochemical analysis. The roots were surface sterilized as follows: roots were washed thoroughly with tap water to remove attached soil particles, immersed in 1% Tween 20 for 2 min, 70% ethanol for 5 min, and 2% NaClO for 20 min and washed 5 times with sterile distilled water. This procedure has been successfully tested for barley [22]. After this procedure, the roots were frozen in liquid nitrogen and stored at −80 °C until further DNA extraction procedures.

### 2.3. Peroxidase Activity in Barley Leaves

Leaves were grounded in liquid nitrogen using sterile mortar and pestle. Peroxidase activity, as an indicator for plant stress, was measured according to Schröder et al. [23]. Three grams of the fine powder were homogenized with 30 mL extraction buffer (pH 7.8) containing 0.1 M Tris, 5 mM ethylenediaminetetraacetic, 1% polyvinylpyrrolidone K90, 1% Nonidet P 40 and 5 mM dithioerythritol at 4 °C for 30 min. After centrifugation at 20,000× *g* for 30 min at 4 °C, samples were filtered through a Miracloth filter (Merck Millipore, Darmstadt, Germany). The supernatant was precipitated progressively by adding ammonium sulfate in two subsequent steps. First, ammonium sulfate was added to the supernatant up to a concentration of 40% (*w*/*v*) and the mixture was centrifuged at 20,000× *g* for 30 min at 4 °C. After centrifugation, the supernatant was transferred to a clean beaker and ammonium sulfate was added up to a concentration of 80% (*w*/*v*). The mixture was then centrifuged at 20,000× *g* for 30 min at 4 °C again. Afterwards, the supernatant was discarded carefully. The obtained pellet was re-suspended in 2.5 mL of 25 mM Tris-HCl buffer (pH = 7.8). Proteins were desalted by chromatography through PD 10 columns (GE Healthcare, Buckinghamshire, UK) and stored at −80 °C for further use. Peroxidase (POX, EC 1.11.1) activity was assayed at 420 nm using 4 M guaiacol as the substrate and H_2_O_2_ (2.56 M per reaction).

### 2.4. DNA Extraction and Sequencing Library Preparation

DNA was extracted from surface sterilized roots (4 pots per treatment, *n* = 4). Therefore, roots were ground in liquid nitrogen; 0.1 g of ground material was used for extraction of nucleic acids according to Griffiths et al. [24], using bead beating (Precellys24 Tissue Homogenizer, Montigny-le-Bretonneux, France) for extraction and a phenol-chloroform based purification. The primer pair 335F/769R [25] was used for the bacterial 16S rRNA gene amplification. For fungi, a mixture of 5 forward primers for Internal transcribed spacer (ITS2) and a mixture of 4 reverse primers for ITS2 and full ITS [26] were used for better coverage [27]. All primers were fused with Illumina adaptors. The reaction mixture contained 2.5 µL NEB Next High Fidelity Master Mix (Illumina, San Diego, CA, USA), 0.5 µL of each primer (10 pmol/µL), 2.5 µL PCR additives, 100 ng template DNA and water treated with 0.1% diethyl pyrocarbonate to a final volume of 25 µL. Bovine serum albumin (BSA) and 36 mM tetramethylammonium were used as PCR additives for 16 S rRNA and ITS amplification, respectively. The PCR cycling conditions consisted of an initial denaturation step of 98 °C for 2 min, followed by 30 cycles involving 10 s of denaturation at 98 °C, 30 s of annealing at 60 °C or 61 °C (16S RNA and ITS, respectively) and 30 s of extension at 72 °C, with a final extension at 72 °C for 5 min. Triplicate PCR reactions were pooled and purified using Agencourt AMPure XP kit (Beckman Coulter, Brea, CA, USA). The purified products were quantified using the Quant-IT PicoGreen dsDNA assay kit (Life Technologies Europe, Gent, Belgium). Equimolar concentrations of the barcoded amplicons samples were prepared and diluted to a final concentration of 4 nM. The library was sequenced using an Illumina Miseq platform (Illumina, San Diego, CA, USA). The obtained sequencing data has been deposited at NCBI under the accession number PRJNA629446.

### 2.5. Bioinformatic Analysis

The raw sequences were analyzed with QIIME (v. 1.9.1) [28]. Adaptors and primers were removed using AdapterRemoval [29]. Phix contaminations were removed using the program Deconseq [30]. Reads were merged and filtered by size (amplicon length) and quality (Phred quality score > 2). The sequences were then clustered into operational taxonomic units (OTUs) using open reference strategy based on 97% similarity. Bacterial reads were assigned with the SILVA 132 SSU database (https://www.arb-silva.de/documentation/release-132/ accessed on 9 April 2020). OTUs assigned to chloroplast were filtered out with the command filter_taxa_from_otu_table.py. 

ITS sequences were extracted using ITSx (v. 1.0.11) [31]. This step ensured that conserved SSU, 5.8S regions, and ITS chimeric sequences were removed, resulting in a reliable operational taxonomic unit (OTU) clustering [32]. The resulted ITS2 sequences were then used for OTU clustering with an open reference strategy based on 97% similarity. UNITE database (v. 7.1) was used as reference [33]. Taxonomy was assigned with RDP classifier (v. 2.2) [34]. OTUs assigned to Plantea were filtered out with the command filter_taxa_from_otu_table.py.

### 2.6. Statistical Analysis

The statistical analyses were performed in R (v. 3.6.2). Significant differences in alpha diversity were evaluated with *t*-test. Unifrac distances and principal coordinate analyses were used to construct and visualize the dissimilarity matrices based on the bacterial and fungal community composition. Permutational multivariate analysis (PERMANOVA) was conducted to test the significance of the a priori classified groups, using the function “adonis” from the R package “vegan”. Results with *p*-value below 0.05 were considered statistically significant. A biomarker detection pipeline linear discriminant analysis (LDA) effect size (LEfSe) [35], available at http://huttenhower.sph.harvard.edu/galaxy/ (accessed on 9 April 2020), was used to detect which microbial taxa were enriched in each group. As a first step, the nonparametric factorial Kruskal-Wallis (KW) rank sum test was used to detect taxa with significant differential abundances. Biological consistency was subsequently investigated with a set of pairwise tests among subclasses using the (unpaired) Wilcoxon rank sum test. Finally, LDA was used to estimate the effect size of each differentially abundant trait. Alpha values of 0.05 were used for the KW rank sum test, and a threshold of 2.0 was chosen for logarithmic LDA scores.

The differences in POX activity and plant dry mass between treatments were compared with *t*-test, after checking for normal distribution using the Shapiro-Wilks test and Q-Q plot.

## 3. Results

### 3.1. Effects of Drought on Plant Performance in Natural and Disturbed Soils

As expected, the water content of soil was affected by the drought treatment significantly (*p* < 0.05). At T1, we detected 12.18 ± 0.87% in NSM and 9.23 ± 1.20% in DSM under regular watering to 3.14 ± 0.84% and 3.13 ± 0.26% under drought stress in NSM and DSM, respectively (Figure S1). At that time point, leaves from stressed plants had approximately one-tenth water content compared to plants grown in control soils, independent of the status of the soil microbiome (Figure S1). Moreover, plants subjected to drought stress exhibited visible signs of water deficit, as they showed leaf rolling and the color of leaves turned from green to yellow. 

**Figure 2:**
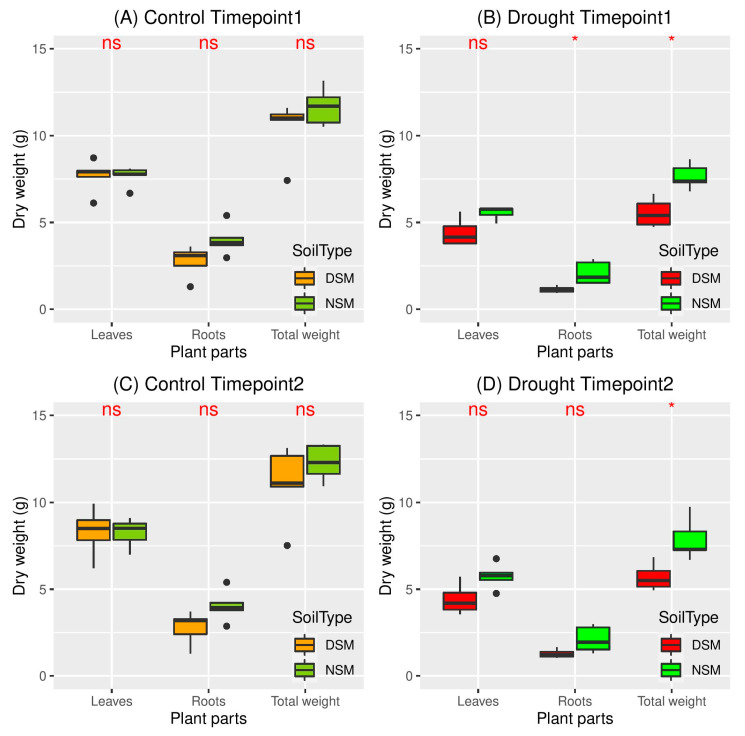
Plant biomass of barley grown in DSM (soil with disturbed microbiome) and NSM (soil with natural microbiome) under regularly watering (orange and olive green) and drought (red and green) at the timepoint 1 (11 days after drought: **A**,**B**) and timepoint 2 (2 weeks after re-watering: **C**,**D**) (*n* = 5) (ns: not significant; *: significant, *p* < 0.05).

No significant differences between NSM and DSM were observed for the water content of the leaves when plants regularly received water. Similarly, when regularly watered, the dry mass of plants grown in NSM and DSM did not differ significantly. However, under water limitation, dry mass values were significantly higher for plants grown in NSM compared to DSM (*p* < 0.05). These differences mainly resulted from the lower root biomass detected for the drought-stressed plants in DSM, as significant differences between NSM and DSM (*p* < 0.05) were observed only for roots. Two weeks after drought alleviation, plant dry mass values remained higher for plants grown in NSM soil compared to DSM soil (Figure 2).

We further used the ROS scavenging enzyme, peroxidase (POX), as an indicator for the redox status under stress. We detected significant higher POX activity in the leaves of drought stressed plants compared to regularly watered plants (*p* < 0.05), independent of the soil (Figure S2). Moreover, we evaluated how plants recover from stress in both soils. Two weeks after drought alleviation, POX levels remained high in plants grown in the DMS, whereas we found a trend towards a lower POX activity for plants grown in the NSM soil (Figure S2). 

### 3.2. Effects of Drought on the Diversity of the Root-Associated Microbiome

A total of 3,000,968 16S rRNA and 4,683,223 ITS paired-end reads were obtained. After quality filtering, 2,358,045 16S rRNA and 4,481,449 ITS reads were clustered at 97% sequence similarity level, respectively. OTUs assigned to chloroplast (16S rRNA amplicon) and Plantae (ITS amplicon) were discarded, resulting in 2,233,173 bacterial and 1,430,867 fungal reads. Bacterial reads were rarefied at the sequencing depth of 48,166, while fungal reads were rarefied at the depth of 4002. The rarefaction curves indicated that the sequencing depth was sufficient to represent the diversity of barley root endophytes (Figure S3). 

**Figure 3:**
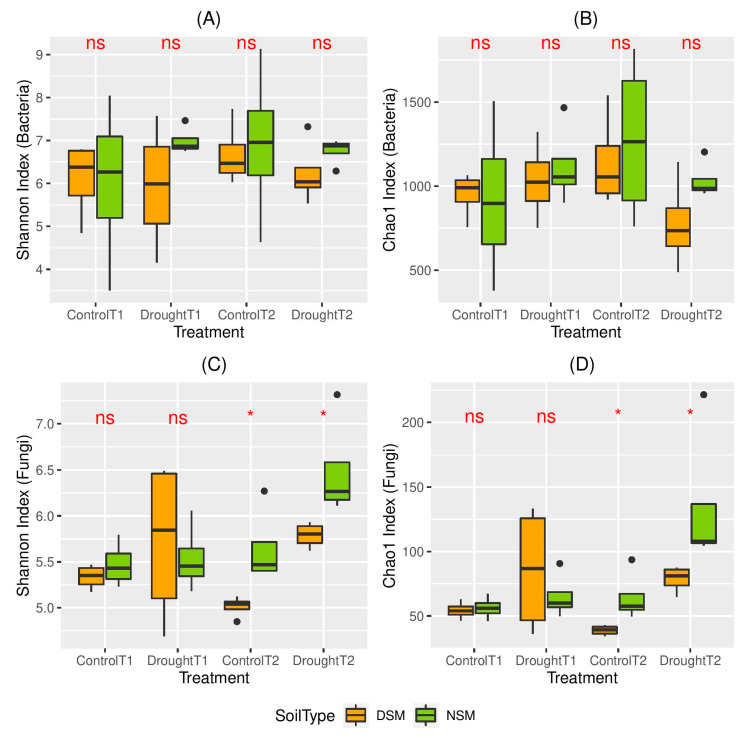
(**A**) Shannon index and (**B**) Chao1 of bacterial root endophytes; (**C**) Shannon index and (**D**) Chao1 index of fungal root endophytes of plants grown in in DSM (soil with disturbed microbiome: orange) and NSM (soil with natural microbiome: green) under control and drought conditions (labeled as D) and control (labeled as C) at timepoint 1 (11 after drought: labeled as 1) and at timepoint2 (After 2 weeks re-watering: labeled as 2). Only the significant groups of comparisons were marked with lines, *: *p* < 0.05.

**Figure 4:**
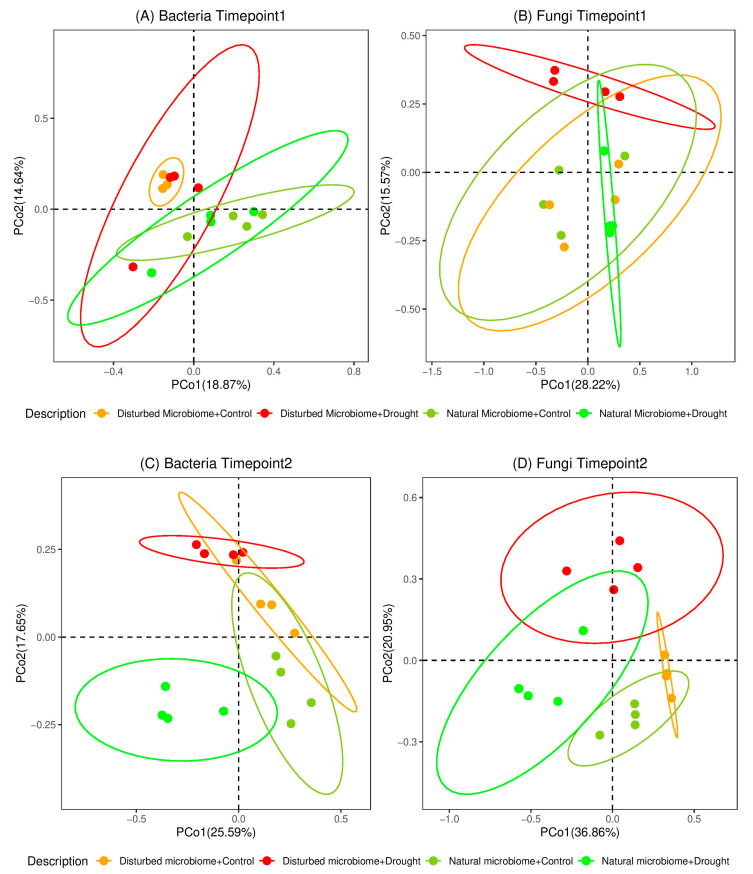
Principal coordinate (PCoA) plots of root endophytic (**A**) bacteria and (**B**) fungi at T1, (**C**) bacteria and (**D**) fungi at T2 in DSM and NSM (*n* = 4). The unweighted Unifrac metric was used for bacteria while Bray-Curtis dissimilarity was applied for fungi. The ellipses represent 95% confidence interval of corresponding samples.

We compared the diversity and richness of bacterial and fungal endophytic communities calculated as Shannon diversity index and Chao1 index. At T1, the alpha diversity of bacterial and fungal endophytes was influenced neither by the pre-treatment of the soil nor by the water regime (Figure 3). We further performed permutational multivariate analysis to evaluate the impacts of drought stress and the status of the soil microbiome on the composition of the endophytic bacterial and fungal communities (Figure 4). As expected, the overall composition of bacterial communities significantly differs in NSM and DSM soils. In accordance, two major clusters based on the microbiome status of the two soils were observed in the principal coordinate (PCoA) plots (Figure 4A). However, the differences of fungal communities driven by the status of the soil microbiome were only observed under drought conditions. Surprisingly, directly after the drought period, a significant influence of the drought stress was neither observed for NSM nor for DSM for both bacterial and fungal communities. 

For T2, no differences of α-diversity were observed for bacterial communities. In contrast, higher diversity and richness of fungal communities were detected in the roots of drought-alleviated plants compared to control plants. However, this was proven significant for DSM (*p* < 0.05) (Figure 3). Moreover, the data showed a higher α-diversity of the fungal communities of the drought-alleviated plants compared to drought-stressed plants in NSM (T2 versus T1), whereas no significant differences were observed for DSM. (Figure 3). Permutational multivariate analyses showed that, at T2, the composition of bacterial and fungal communities was affected by both factors, the changes in water regimes as well as the original soil microbiome (Figure 4). This reflects changes in the composition of microbial communities from drought-stressed plants that occurred two weeks after re-watering. Interestingly, changes caused by drought treatment were significant for fungal and bacterial communities associated with plants grown in NSM, but not for DSM (Figure S4).

### 3.3. Effects of Drought on Endophytic Bacteria

**Figure 5:**
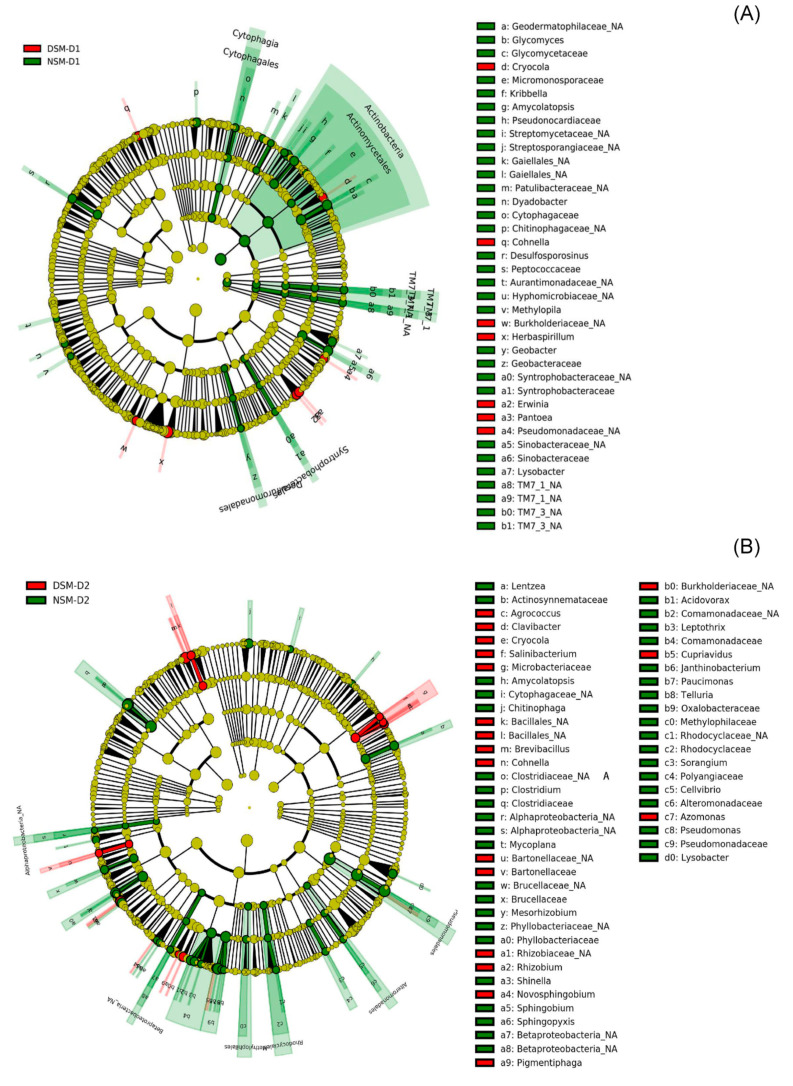
The cladogram showing differentially abundant taxa as biomarker in the bacterial root endophytes under drought. The biomarker was identified with Linear Discriminant Analysis (LDA) Effect Size (LEfSe) using Kruskal-Wallis test (*p* < 0.05) with LDA score >2 (*n* = 4). (**A**) Biomarker taxa identified in NSM (soil with natural microbiome) and DSM (soil with disturbed microbiome) directly after drought stress. (**B**) Biomarker identified in NSM and DSM 2 weeks after re-watering.

Proteobacteria was by far the most abundant phylum, regardless of the treatment, followed by Firmicutes and Actinobacteria, representing 68.1%, 20.6%, and 7.3% of the total number of reads. At genus level, Pseudomonas, Agrobacterium, and Stenotrophomonas were the most abundant bacterial endophytes detected in barley roots at T1 (Figure S5A). We used LEfSe to detect features characterizing the differences between NSM and DSM soils under drought stress. Most of the taxa differentially abundant in NSM soil belong to the phylum Actinobacteria, including Glycomycetaceae (Glycomyces), Micromonosporaceae, Pseudonocardiaceae (Amycolatopsis), Streptomycetacea, Streptosporangiaceae, and Geodermatophilaceae. Moreover, taxa assigned to the candidate division TM7, recently renamed to Saccharibacteria, Proteobacteria from the family Syntrophobacteraceae (unclassified) as well as Sinobacteraceae and Bacteroidetes from the family Cytophagaceae (Dyadobacter) were identified for NSM. In contrast, 5 out of 7 taxa identified as characteristic for DSM were members of the phylum Proteobacteria. Herbaspirillum, Erwinia, Pantoea, and an unclassified Pseudomonaceae showed high LDA score (Figure 5A). 

We further investigated changes in endophytic bacterial communities two weeks after the drought stress was ceased. In T2, Pseudomonas, Stenotrophomonas, Agrobacterium, Achromobacter, Rhizobium, and Burkholderia were the major bacterial genera detected in the roots of barley plants (Figure S5B). Mainly taxa assigned to the phyla Proteobacteria and Firmicutes were characteristic for plants grown in NSM soil after drought stress alleviation. Among these, Pseudomonas, Janthinobacterium, Clostridium, Mesorhizobium, and unclassified Phyllobacteriaceae and Comamonadaceae showed high LDA scores. Taxa from the phyla Proteobacteria and Firmicutes were also characteristic for plants growing in DSM. However, Rhizobium, Cohnella, Brevibacillus, Azomonas, and unclassified Rhizobiaceae and Burkholderiaceae were identified for this soil (Figure 5B).

### 3.4. Effects of Drought on Endophytic Fungi

**Figure 6:**
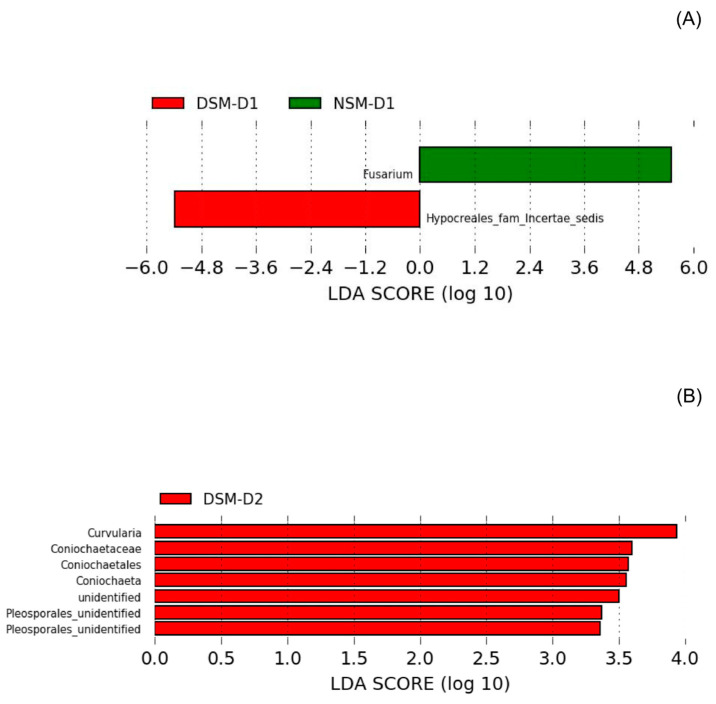
Linear Discriminant Analysis (LDA) Effect Size (LEfSe) plot of differentially abundant taxa as biomarkers for the fungal root endophytes under drought determined using Kruskal-Wallis test (*p* < 0.05) with LDA score >2 (*n* = 4). (**A**) Biomarker taxa identified in NSM (soil with natural microbiome) and DSM (soil with disturbed microbiome) directly after drought stress. (**B**) Biomarker identified in NSM and DSM 2 weeks after re-watering.

For all treatments, the majority of fungal OTUs (52% ± 3%) were assigned to the phylum Ascomycota. The genera Fusarium, Gibberella, Setophoma, and Sarocladium were the most abundant in NSM, whereas DSM Trichoderma, Alternaria, Cladosporium, Penicillium, and Microdochium were also detected among the most dominant endophytic fungi (Figure S6). Due to the higher variability among replicates observed for fungi analyses, we observed fewer taxa that fulfill the criteria of a biomarker set for the LEfSe analyses. At T1, we observed that *Fusarium* and an unclassified Hypocreales fam incertae sedis were identified as characteristic for plants grown under drought stress in NSM and DSM soil, respectively (Figure 6A). 

We further investigated changes in the composition of fungal endophytes two weeks after drought stress alleviation (T2). Fusarium, Chetomium, Trichoderma, Gibberella, Setophoma, and Microdochium were the major groups in endophytic fungi of barley roots. Fungal biomarkers for drought-alleviated plants were only identified for DSM soil and all belonged to the phylum Ascomycota. Among those, the genera Curvularia and Coniochaeta showed high LDA scores (Figure 6B). 

## 4. Discussion

### 4.1. Drought Stress Differently Affect Plant Growth in Soils with Natural and Disturbed Microbiome

As expected, plants submitted to drought stress exhibited visible signs of water deficit, including lower dry mass, particularly for the roots. However, dry mass values were higher for plants grown in NSM compared to DSM. This might be linked to root microbiome composition, as the overall composition of both bacterial and fungal communities was clearly influenced by soil status. Differences in β-diversity were expected, as the soil microbiota is considered the major driver of root associated microbial communities [36,37]. Hence, it is expected that differences in the composition of the soil microbiome will be reflected in the root microbiome. However, we did not observe effects of soil pretreatment and diversity of root endophytes. This was unexpected, as we observed a clear reduction of the microbial biomass, as assessed by DAPI staining. Although we were aware that soil sterilization process is not 100% efficient, as also discussed elsewhere [38], it is well known that soil microbial communities are strongly disturbed during this process [39,40]. However, barley seeds possess a very diverse microbiome [22,41,42]. Therefore, even though the soil microbiome was affected, seed-borne endophytes might have occupied the free niche in root tissue and, hence, no significant differences were observed when α-diversity was considered.

After 11 days of water limitation, we detected high POX activity levels for plants grown in both soils. Increases in activity of ROS scavenging enzymes as a response to drought stress in cereals have been extensively documented [43,44]. POX levels remained high in plants grown in DSM two weeks after re-watering, whereas a trend to lower POX activity for plants grown in NSM was observed. This would suggest reduced ROS stress as a possible mechanism for better response in NSM. We assume that soil microbes helped the plants to cope with the stress, by modifying its physiological response. For example, it was shown that the fungus *Piriformospora indica* can modulate metabolic response of barley plants to water deficit, mainly by inducing changes in the abundance of proteins which are involved in the plant’s primary metabolism, in particular, acting to mitigate the damage caused by oxidative stress [45]. 

### 4.2. The Diversity of the Root-Associated Microbiome Did Not Change Directly After Drought Stress 

Drought did not significantly affect the diversity and richness of bacterial and fungal endophytes, though plants showed clear signs of water stress. Yet the richness and diversity of soil microbial communities can be strongly affected by drought, as shown by de Vries et al. [46]. This probably reflects the differences in the habitats, as microorganisms living inside or tightly associated with plants benefit from mechanisms used by the host to respond to water deficit, whereas those in living in the soil are more directly affected by the stress. Therefore, microbial communities might change more rapidly in the soil than inside the plant. 

### 4.3. Enrichment of Soil Microbes in the Roots of Drought Stressed Plants 

We predicted a higher relative abundance of Actinobacteria in the roots of drought-affected plants, as shown in different studies assessing the effects of drought on the composition of bacterial communities associated with the roots of grasses [47,48]. In our study, we did evaluate changes in relative abundance and, hence, cannot discard the possibility that the absolute numbers of total Actinobacteria increases, even though those are not reflected in relative abundances. Nevertheless, we detected a significant increase in the abundance many Actinomycetes taxa in the roots of drought-stressed plants grown in NSM soil. Moreover, many OTUs assigned to Actinobacteria were identified as indicators of drought stress for NSM but not for DSM the disturbed soil. As shown by Yang et al. (2017), Actinobacteria that colonize the roots of different barley cultivars are mostly derived from the soil and are not a major part of the seed-derived microbiome. This was likewise the case for Deltaproteobacteria and Bacteroidetes, for which we also detected higher abundances in plants grown in natural soils but not for those grown in the soils with disturbed microbiome. Whether the bacteria enriched in drought-stressed plants do positively affect their hosts or just benefit from free niches usually occupied by other organisms when plants are not water limited, still needs to be investigated. 

Furthermore, many taxa from the phylum Saccharibacteria (former candidate division TM7) were identified as biomarkers for NSM soils. This result was intriguing, as Saccharibacteria were described as ultra-small parasitic bacteria that live in tight association with Actinobacteria [49,50] and was also reported to be enriched in drought-affected maize plants [51]. Besides, Saccharibacteria were shown to degrade salicylic acid [50], a phytohormone involved in the regulation of drought stress response [52], which also modulates the root microbiome assembly [53].

Soil fungi are in general considered to be more resistant to drought than bacteria, at least in short term periods such as in the present study [46]. In fact, our data indicates that endophytic fungal communities were less affected directly after drought. Only one Fusarium and an unclassified Hypocreales fam incertae sedis were identified as characteristic biomarker for the plants grown in NSM and DSM soils and submitted to drought stress, respectively. It is hard to make any conclusion at this taxonomic level, particularly in the case of barley, for which functional traits rather phylogenetic affiliation seems to be determinant for colonization [54]. 

### 4.4. Seed-Borne Organisms Were the Major Drought Responders in Soils with Disturbed Microbiome

Many bacterial OTUs shown to be differentially abundant in the roots of plants submitted to drought stress were assigned to genera, frequently detected in barley seeds, namely Pantoea, Erwinia, and Pseudomonas (Rahman et al., 2018; Yang et al., 2017). This holds particularly true for plants grown in the soil with disturbed microbiome, reinforcing the idea that under these conditions a higher proportion of the root microbiome is originated from the seed-borne microbiome. Many of these bacterial strains from these genera were shown to improve the response of plants to stress. This is the case for seed-borne Pantoea strains that primed barley immune response to the pathogen Blumeria graminis (Rahman et al., 2018) and promoted considerable growth of wheat seedlings under saline stress [55]. Besides, Pantoea strains might also improve plant fitness under drought stress [56].

For the fungal communities, we observed an increase in the abundance of genera normally associated with pathogenesis in barley plants, such as Gibberella and Gaeumannomyces. This result was intriguing as there were no signs of pathogenesis throughout the experiment. Even though we cannot discard that these were truly fungal pathogens, which colonized the host without causing symptoms, it is also possible that they are seed-borne endophytes. Geisen et al. (2017) have shown that most seed endophytes mostly resembled pathogens. Interestingly, in this study, they observed no overlap between the root and seed endophytes, mainly when plants were grown in non-sterile soil. Anyhow, differentiation between pathogenic and non-pathogenic strains from the same fungal species is difficult and relies on other markers than ITS sequences (Lievens et al., 2008), used in the present study. Furthermore, according to Redman [57] the host physiology is likely to control the lifestyle of the colonizing fungus.

### 4.5. Changes in the Composition of Root-Associated Endophytes from Drought Alleviated Plants 

There is evidence for a different allocation of plant carbon to soil fungi and bacteria during drought and at the recovery phase [58], which could have influenced the recruiting of soil microorganisms. According to Fuchslueger, drought might weaken the link between plant and bacterial, but not fungal, carbon turnover, and facilitate the growth of potentially slow-growing, drought-adapted soil microbes, such as Gram-positive bacteria. This corroborates our findings, in which many taxa of the phylum Firmicutes were identified as biomarkers for the drought settings in both NSM (Clostridium) and DSM soils (Brevibacillus). Moreover, we detected a general increase in diversity for fungi, particularly in DSM soil. 

Interestingly, we only identified fungal biomarkers for DSM soils, namely Curvularia and Coniochaeta. The first was described to form symbiotic associations with Dichanthelium lanuginosum, thereby increasing plant host tolerance to high soil temperatures [59]. High abundances of Curvularia were also observed in grassland soils under to water deficit [60]. Coniochaeta sp. were classified according to their origin, namely as soil, dung or wood originated, with a few cosmopolite species. Among the soil forms, several are common in post-fire soils [61]. These fungal taxa could better survive the harsh treatment to which the DSM soil was submitted. Effects of Curvularia and Coniochaeta on the barley plant performance under drought stress should be addressed in future experiments. 

## 5. Conclusion

Soil holds an extremely high diversity of microorganisms, taxonomic and functional, which might be essential for the plants to cope with a continuously changing environment (flooding, drought, different types of pathogens, heat, etc.). In the present study, we have demonstrated the relevance of soil microorganisms for the response of plants to drought stress. Moreover, we identified many microbial groups with potential beneficial effects for the growth of barley plants when submitted to stress. Knowledge on the mechanisms by which soil microbes interfere with the response of plants to abiotic stressors is essential and should be implemented in future studies. This could benefit future breeding approaches, for example, by the selection of plants with higher phenotypic plasticity due to the interaction with the soil microbiome. 

## Supplementary Materials

The following are available online at https://www.mdpi.com/2076-2607/8/9/1414/s1, Figure S1: Relative water content of (A) soil and (B) leaves in DSM and NSM under different treatments at sampling time T1 and T2 (*n* = 5). Figure S2: Peroxidase activity in leaves of barley plants grown in in DSM (soil with disturbed microbiome) and NSM (soil with natural microbiome) under control, drought and Re-watered conditions (*n* = 5). Figure S3: Rarefaction curves of root endophytic (A) bacteria and (B) fungi (*n* = 4). Figure S4: PCoA plots of root endophytic bacteria in NSM using (A) weighted (B) unweighted Unifrac distances and in DSM using (D) weighted and (E) unweighted Unifrac distances under drought and re-watered condition. PCoA plots of root endophytic fungi using Bray-Curtis dissimilarity in (C) NSM and (F) DSM under drought and re-watered condition (*n* = 4). Figure S5: Major genera of bacterial endophytes in roots grown in NSM and DSM at (A) T1 and (B) T2 (*n* = 4). Figure S6: Major genera of fungal endophytes in roots grown in NSM and DSM at (A) T1 and (B) T2 (*n* = 4).
