# Supplementary material for: Response of Barley Plants to Drought Might Be Associated with the Recruiting of Soil-Borne Endophytes
Source: Microorganisms. 2020 Sep 14;8(9):1414. doi: 10.3390/microorganisms8091414 (PMC7565417; doi:10.3390/microorganisms8091414)
Supplement: Supplementary file 1 [file microorganisms-08-01414-s001.pdf]

Supplementary table 1: 16S rRNA and ITS primers and PCR conditions

rRNA primer set	seq 5'->3'		
335f	CADACTCCTACGGGAGGC		
769r	ATCCTGTTTGMTMCCCVCRC		
**Reference**			
Dorn et al 2015			
**Reagent**	**Stock concentration**	**Final concentration**	**µl /tube**
**H2O**			
**NEB Next High Fidelity Master Mix**	2x	1x	12.5
**335f**	10 pmol.µl-1	5 pmol	0.5
**769r**	10 pmol.µl-1	5 pmol	0.5
**BSA**	15 µg.µl-1		2.5
**sample**			
**Set**	**temperature (°C)**	**time**	
**hotstart**	98	2 min	
**denaturation**	98	10 s	
**annealing**	60	30 s	
**elongation**	72	30 S	x 30
	72	5 min	
**ITS primers**	**seq 5'->3' (forward-reverse combinations)**		
ITS3-mix*			
ITS3-Mix1 (Fungi)	TCGTCGGCAGCGTCAGATGTGTATAAGAGACAG-CATCGATGAAGAACGCAG		
ITS3-Mix2 (Chytridiomycota)	TCGTCGGCAGCGTCAGATGTGTATAAGAGACAG-CAACGATGAAGAACGCAG		
ITS3-Mix3 (Sebacinales)	TCGTCGGCAGCGTCAGATGTGTATAAGAGACAG-CACCGATGAAGAACGCAG		
ITS3-Mix4 (Glomeromycota)	TCGTCGGCAGCGTCAGATGTGTATAAGAGACAG-CATCGATGAAGAACGTAG		
ITS3-Mix5 (Sordariales)	TCGTCGGCAGCGTCAGATGTGTATAAGAGACAG-CATCGATGAAGAACGTGG		
ITS4-mix*			
ITS4-Mix1 (Fungi)	GTCTCGTGGGCTCGGAGATGTGTATAAGAGACAG-TCCTCCGCTTATTGATATGC		
ITS4-Mix2 (Chaetothyriales)	GTCTCGTGGGCTCGGAGATGTGTATAAGAGACAG-TCCTGCGCTTATTGATATGC		
ITS4-Mix3 (Archaeorhizomycetes)	GTCTCGTGGGCTCGGAGATGTGTATAAGAGACAG-TCCTCGCCTTATTGATATGC		
ITS4-Mix4 (Tulasnellaceae)	GTCTCGTGGGCTCGGAGATGTGTATAAGAGACAG-TCCTCCGCTGAWTAATATGC		
* All primers were diluted to 10 pmol and mixed in equimolar concentration			
**Reference**			
Tedersoo et al 2014 /2015			
**Reagent**	**Stock concentration**	**Final concentration**	**µl /tube**
**H2O**			
**NEB Next High Fidelity Master Mix**	2x	1x	12.5
**ITS3-mix**	10 pmol.µl-1	5 pmol	0.5
**ITS4-mix**	10 pmol.µl-1	5 pmol	0.5
**Tetramethylammonium**	15 µg.µl-1		2.5
**sample**			
**Set**	**temperature (°C)**	**time**	
**hotstart**	98	2 min	
**denaturation**	98	10 s	
**annealing**	61	30 s	
**elongation**	72	30 S	x 30
	72	5 min	

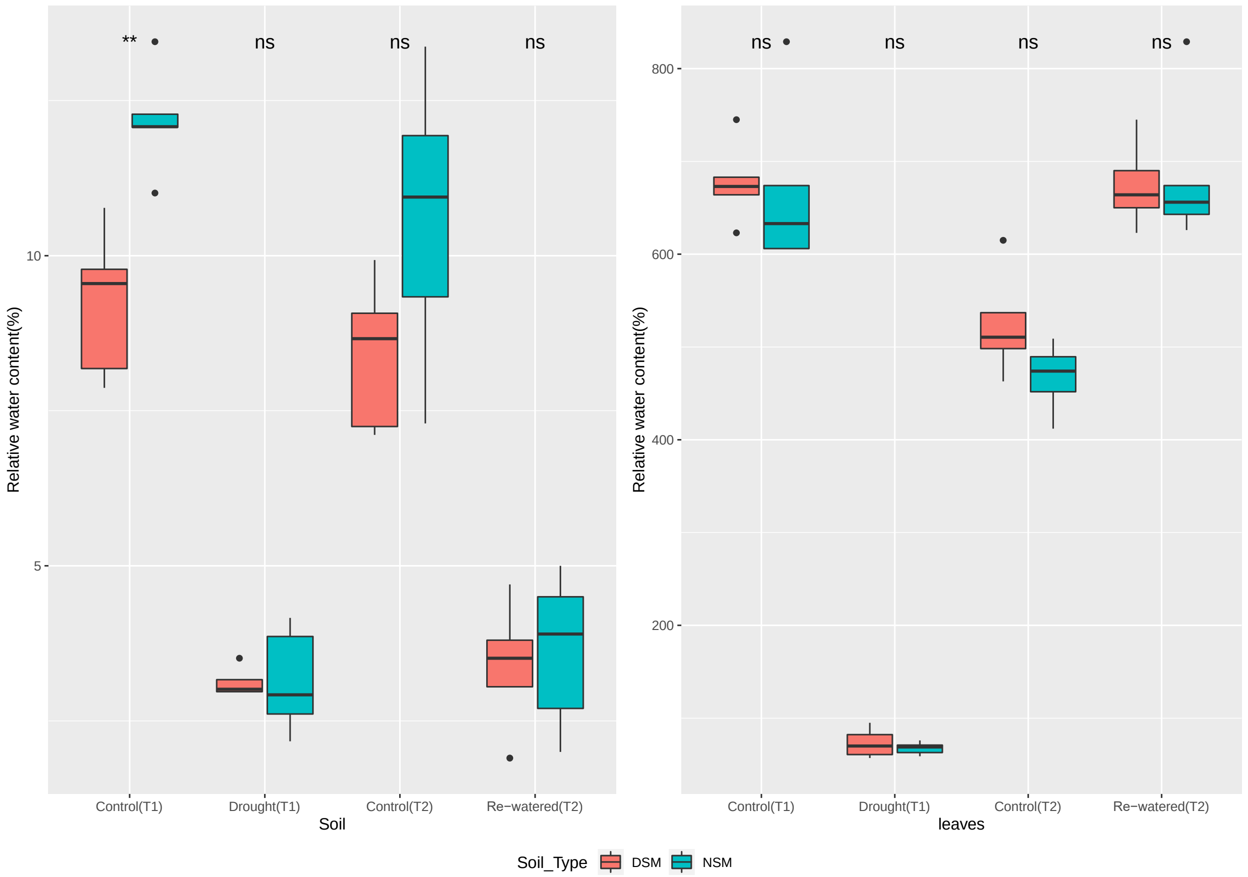

Supplementary Fig. S1 Relative water content of (A) soil and (B) leaves in DSM and NSM under different treatments at sampling time T1 and T2 (n=5). (ns: not significant, **: p<0.01)

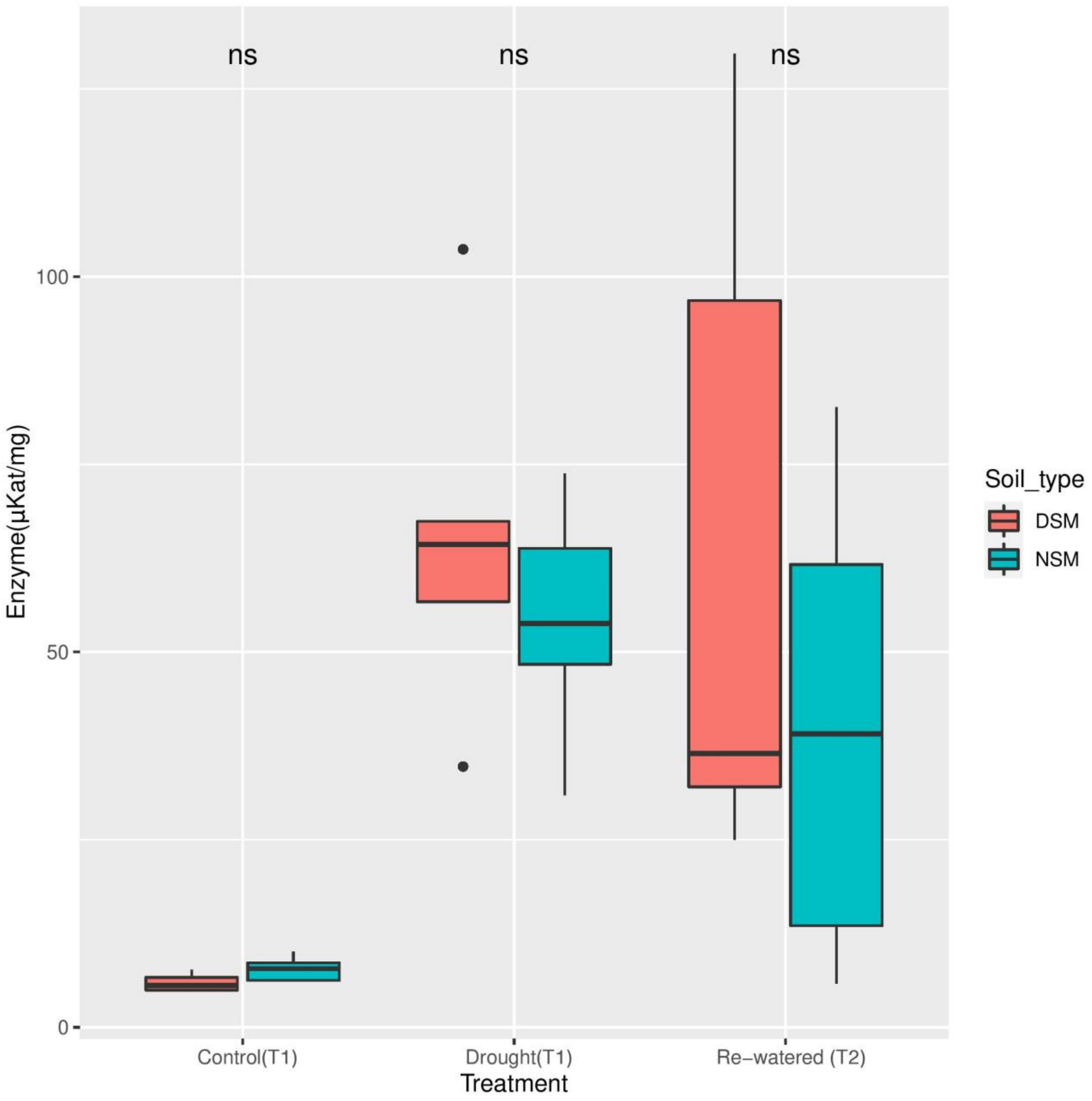

Supplementary Fig. S2 Peroxidase activity in leaves of barley plants grown in in DSM (soil with disturbed microbiome) and NSM (soil with natural microbiome) under control, drought and Re-watered conditions (n=5)

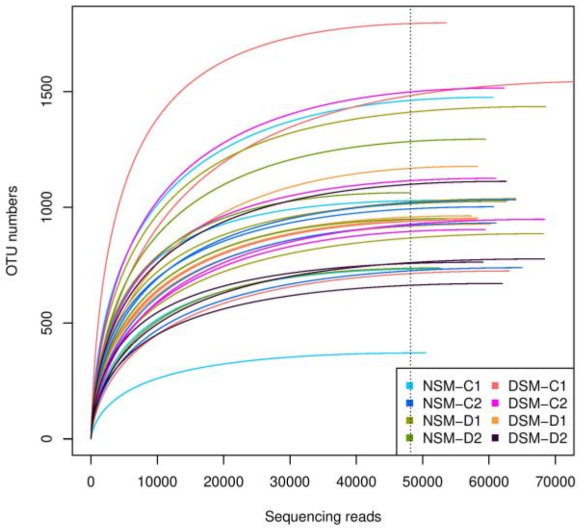

(A)

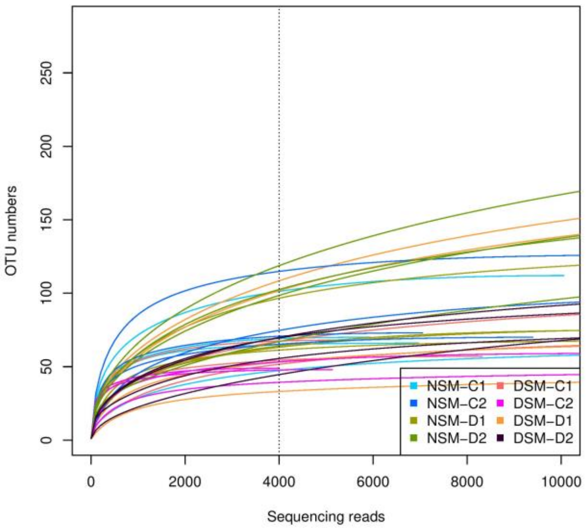

(B)

Supplementary Fig. S3 Rarefaction curves of root endophytic (A) bacteria and (B) fungi (n=4). The bacteria were rarefied at the sequencing depth of 48166 while the fungi were rarefied at the depth of 4002.

(NSM-C1:soil with natural microbiome under regular watering at first sampling;
NSM-C2: soil with natural microbiome under regular watering at second sampling;
NSM-D1: soil with natural microbiome after drought stress;
NSM-D2: soil with natural microbiome drought stressed and re-watered;
DSM-C1: soil with disturbed microbiome under regular watering at first sampling;
DSM-C2: soil with disturbed microbiome under regular watering at second sampling;
DSM-D1: soil with disturbed microbiome after drought stress;
DSM-D2: soil with disturbed microbiome drought stressed and re-watered)

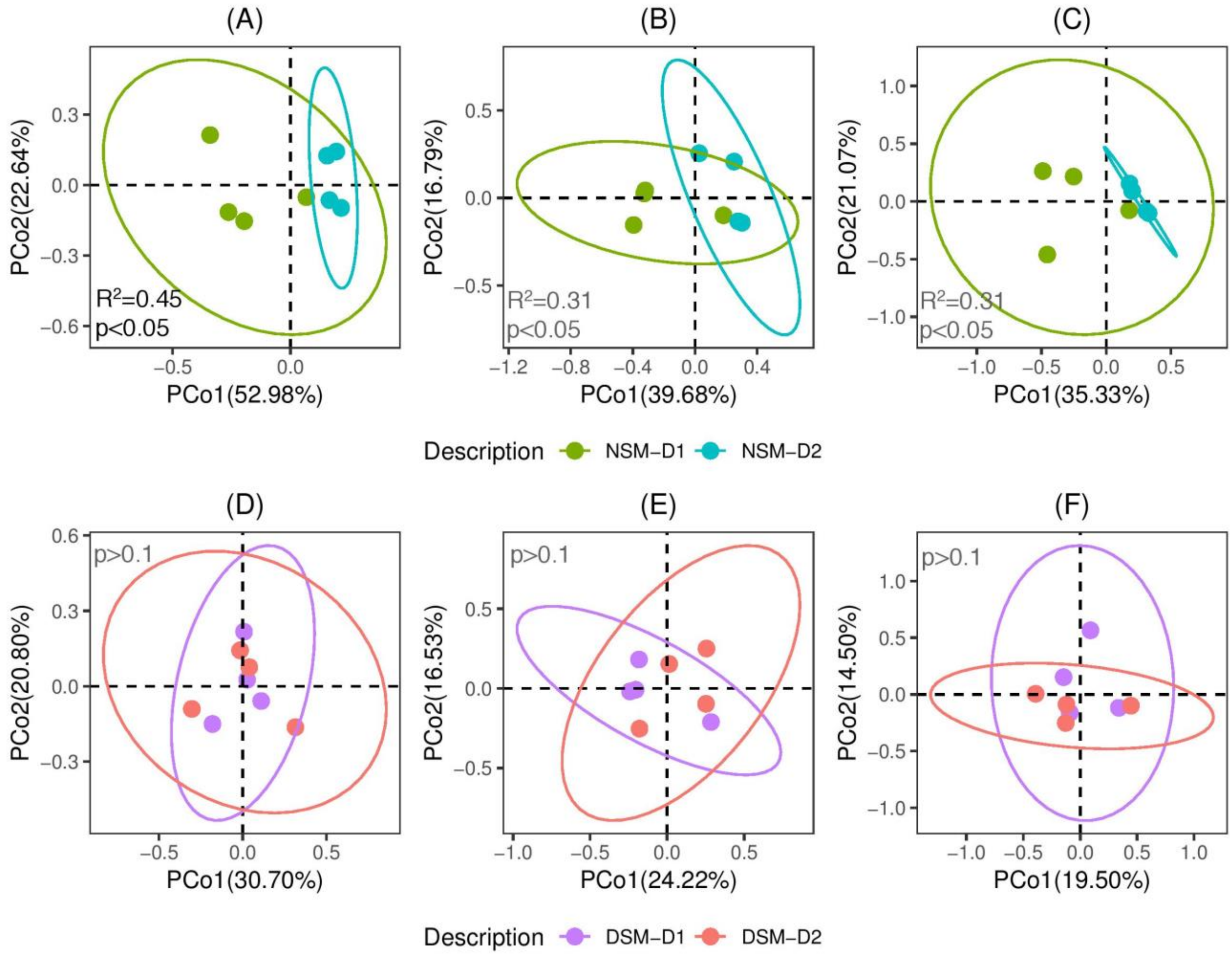

Supplementary Fig. S4 PCoA plots of root endophytic bacteria in NSM using (A) weighted (B) unweighted Unifrac distances and in DSM using (D) weighted and (E) unweighted Unifrac distances under drought and re-watered condition. PCoA plots of root endophytic fungi using Bray-Curtis dissimilarity in (C) NSM and (F) DSM under drought and re-watered condition (n=4). The ellipses represent 95% confidence interval of corresponding samples. (DSM-D1: soil with disturbed microbiome under drought stress; DSM-D2: soil with disturbed microbiome drought stressed and re-watered; NSM-D1: soil with natural microbiome under drought stress; NSM-D2: soil with natural microbiome drought stressed and re-watered)

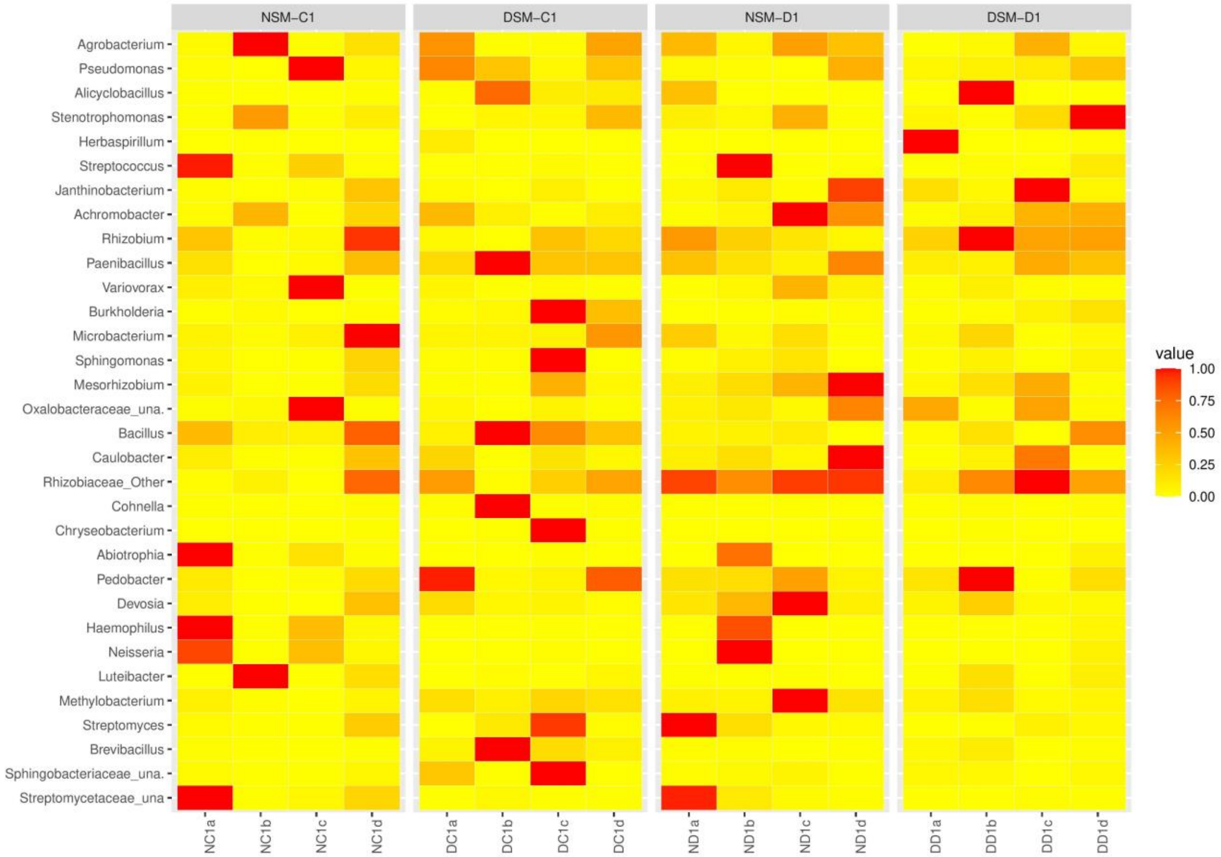
NSM−C1
DSM−C1
NSM−D1
DSM−D1
Agrobacterium
Pseudomonas
Alicyclobacillus
Stenotrophomonas
Herbaspirillum
Streptococcus
Janthinobacterium
Achromobacter
Rhizobium
Paenibacillus
Variovorax
Burkholderia
Microbacterium
Sphingomonas
Mesorhizobium
Oxalobacteraceae_una.
Bacillus
Caulobacter
Rhizobiaceae_Other
Cohnella
Chryseobacterium
Abiotrophia
Pedobacter
Devosia
Haemophilus
Neisseria
Luteibacter
Methylobacterium
Streptomyces
Brevibacillus
Sphingobacteriaceae_una.
Streptomycetaceae_una
NC1a
NC1b
NC1c
NC1d
DC1a
DC1b
DC1c
DC1d
ND1a
ND1b
ND1c
ND1d
DD1a
DD1b
DD1c
DD1d
value
1.00
0.75
0.50
0.25
0.00

(A)

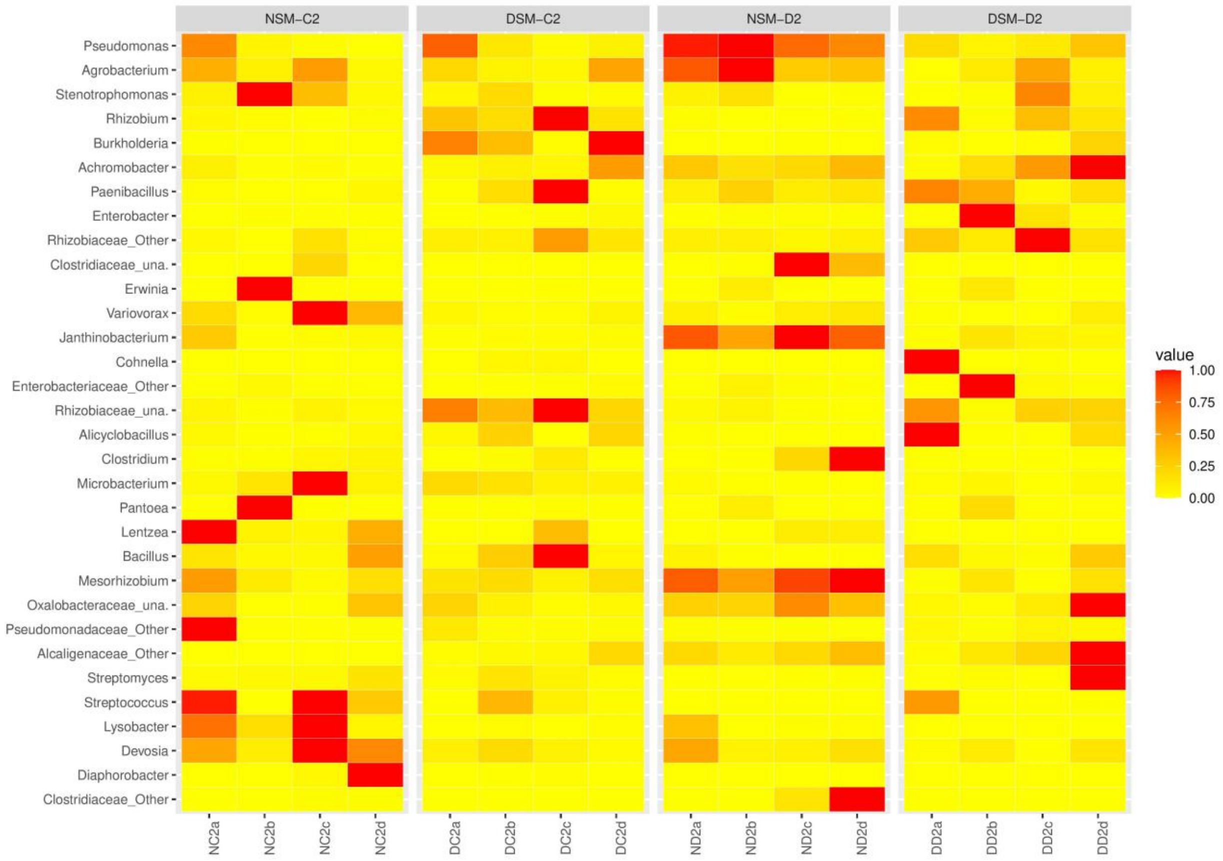

(B)

Supplementary Fig. S5 Major genera of bacterial endophytes in roots grown in NSM and DSM at (A) T1 and (B) T2 (n=4)

(NSM-C1: soil with natural microbiome under control conditions at the first sampling; NSM-D1: soil with natural microbiome under drought stress at the first sampling; DSM-C1: soil with disturbed microbiome under control conditions at the first sampling; DSM-D1: soil with disturbed microbiome under drought stress at the first sampling; NSM-C2: soil with natural microbiome under regular watering at second sampling; NSM-D2: soil with natural microbiome drought stressed and re-watered; DSM-C2: soil with disturbed microbiome under regular watering at second sampling; DSM-D2: soil with disturbed microbiome drought stressed and re-watered)

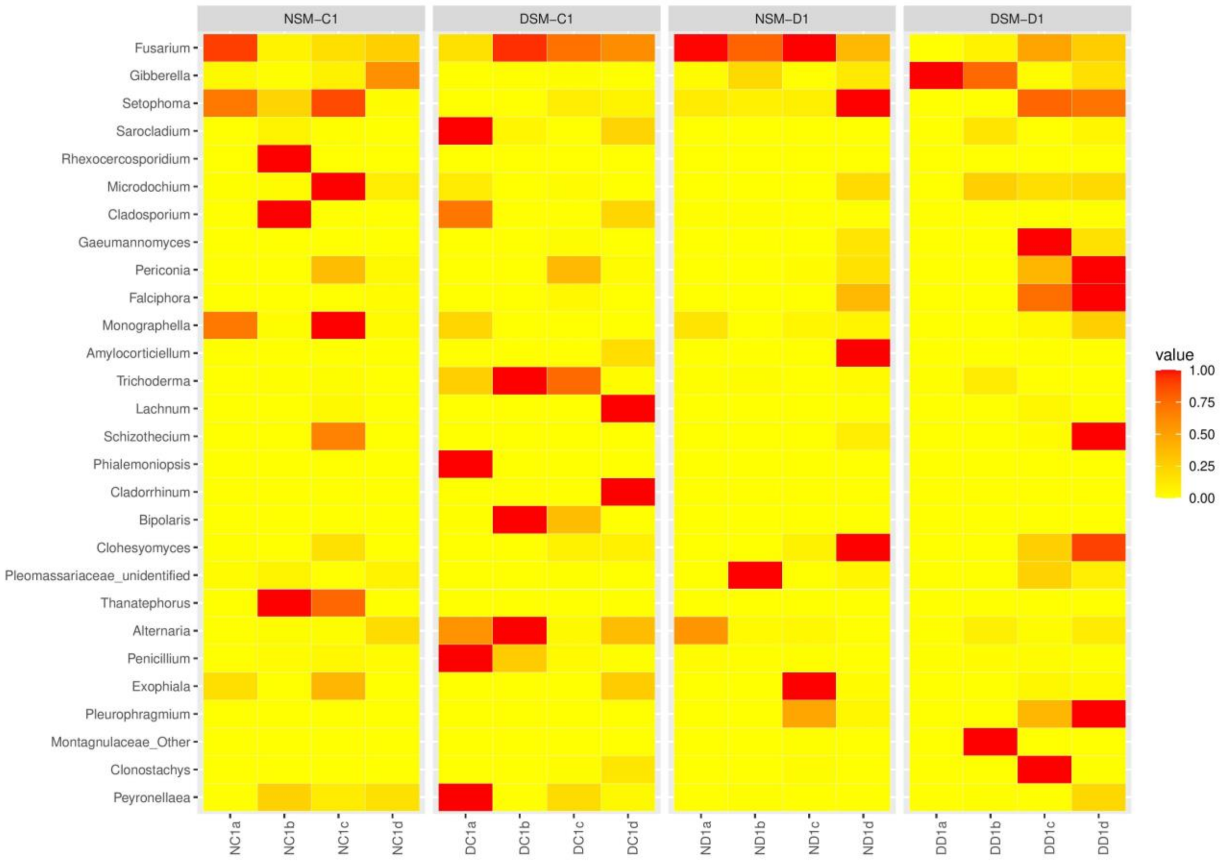

NSM−C1
DSM−C1
NSM−D1
DSM−D1
Fusarium
Gibberella
Setophoma
Sarocladium
Rhexocercosporidium
Microdochium
Cladosporium
Gaeumannomyces
Periconia
Falciphora
Monographella
Amylocorticiellum
Trichoderma
Lachnum
Schizothecium
Phialemoniopsis
Cladorrhinum
Bipolaris
Clohesyomyces
Pleomassariaceae_unidentified
Thanatephorus
Alternaria
Penicillium
Exophiala
Pleurophragmium
Montagnulaceae_Other
Clonostachys
Peyronellaea
NC1a
NC1b
NC1c
NC1d
DC1a
DC1b
DC1c
DC1d
ND1a
ND1b
ND1c
ND1d
DD1a
DD1b
DD1c
DD1d
value
1.00
0.75
0.50
0.25
0.00

(A)

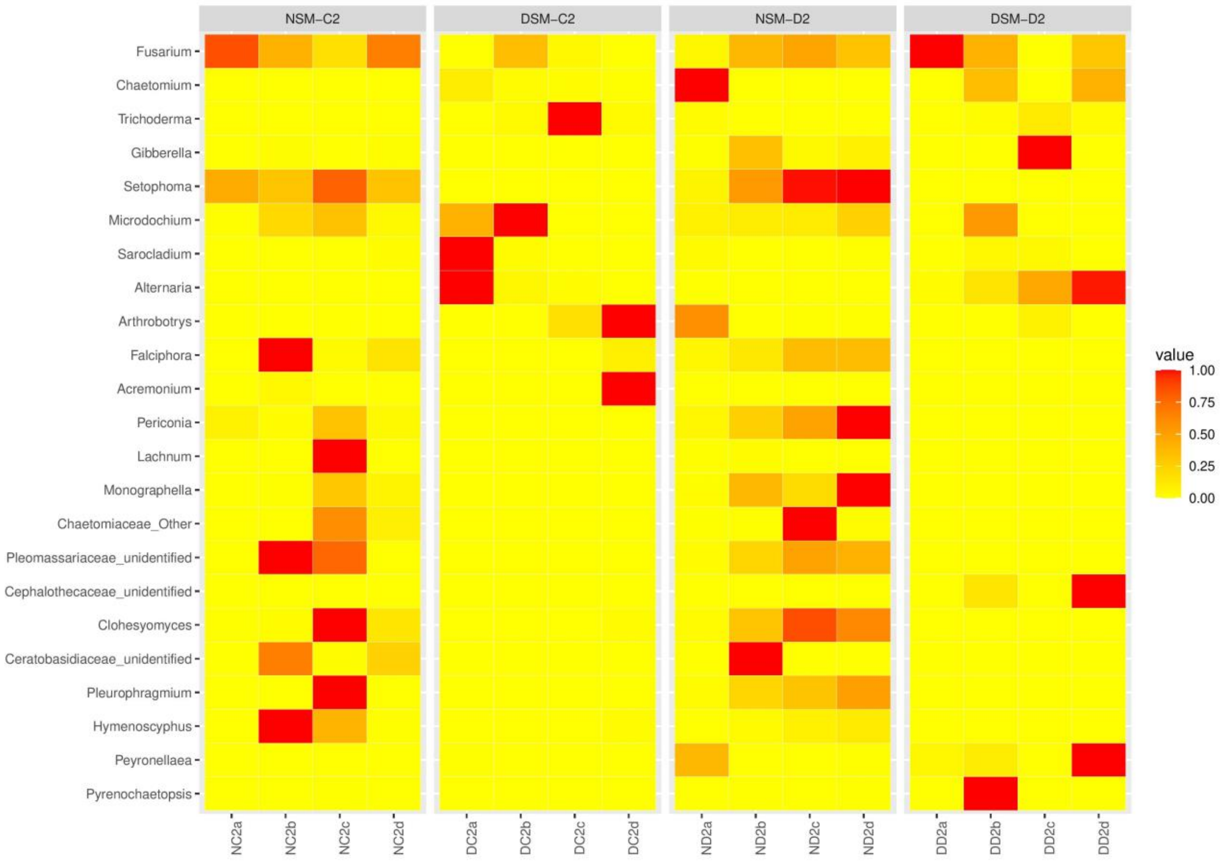

(B)

Supplementary Fig. S6 Major genera of fungal endophytes in roots grown in NSM and DSM at (A) T1 and (B) T2 (n=4)

(NSM-C1: soil with natural microbiome under control conditions at the first sampling; NSM-D1: soil with natural microbiome under drought stress at the first sampling; DSM-C1: soil with disturbed microbiome under control conditions at the first sampling; DSM-D1: soil with disturbed microbiome under drought stress at the first sampling; NSM-C2: soil with natural microbiome under regular watering at second sampling; NSM-D2: soil with natural microbiome drought stressed and re-watered; DSM-C2: soil with disturbed microbiome under regular watering at second sampling; DSM-D2: soil with disturbed microbiome drought stressed and re-watered)
